# Association Between Adherence to the Mediterranean Diet and Asthma in Peruvian Children

**DOI:** 10.1007/s00408-015-9792-9

**Published:** 2015-09-03

**Authors:** Jessica L. Rice, Karina M. Romero, Rocio M. Galvez Davila, Carla Tarazona Meza, Andrew Bilderback, D’Ann L. Williams, Patrick N. Breysse, Sonali Bose, William Checkley, Nadia N. Hansel, GASP Study Investigators

**Affiliations:** Division of Pediatric Pulmonology, School of Medicine, Johns Hopkins University, Baltimore, MD USA; Biomedical Research Unit, A.B. PRISMA, Lima, Peru; Division of Pulmonary and Critical Care, School of Medicine, Johns Hopkins University, Baltimore, MD USA; Department of Environmental Health Sciences, Bloomberg School of Public Health, Johns Hopkins University, Baltimore, MD USA; Program in Global Disease Epidemiology and Control, Department of International Health, Bloomberg School of Public Health, Johns Hopkins University, Baltimore, MD USA

**Keywords:** Peru, Allergic rhinitis, Atopic, Control, Food frequency questionnaire, Diet pattern

## Abstract

**Purpose:**

Adherence to a Mediterranean diet pattern may be associated with lower asthma prevalence in children. We sought to corroborate these findings in Peruvian children.

**Methods:**

This case–control study included children of ages 9–19 years living in Lima, Peru. A food frequency questionnaire (FFQ) was completed and diet pattern was analyzed using a modified Mediterranean diet score (MDS). Primary analysis investigated the relationship between MDS and asthma status. Maternal education, age, sex, and body mass index category were included in multivariate model. Secondary outcomes included asthma control, forced expiratory volume in 1 s (FEV1), allergic rhinitis, and atopic status.

**Results:**

287 participants with asthma and 96 controls without asthma completed a FFQ. Mean age was 13.5 years. According to the asthma control test (ACT), 86 % of those with asthma were controlled (score >19). MDS scores ranged 6–18 (median 15). In adjusted analysis, being above the median MDS scores was associated with decreased odds of asthma [OR = 0.55, 95 % CI (0.33, 0.92), *p* = 0.02]. Among children whose mothers completed secondary education, being above the median MDS significantly decreased the odds of asthma [OR = 0.31, 95 % CI (0.14, 0.71), *p* < 0.01], whereas among those whose mothers did not complete secondary education there was no protective effect [OR = 0.86, 95 % CI (0.43, 1.7), *p* = 0.66]. There was no association between MDS scores and asthma control, FEV1, allergic rhinitis, or atopic status.

**Conclusion:**

Adherence to the Mediterranean diet was inversely associated with having asthma among children in Lima, Peru. This effect was strongest among children with better educated mothers.

## Introduction

Asthma and related allergic diseases are significant public health problems worldwide [1, 2]. Changes in the western diet, which is deficient in antioxidants, are postulated to be one of the reasons for the asthma epidemic [3].

The traditional Mediterranean diet provides food sources rich in antioxidants such as vegetables, legumes, fruits and nuts, cereals, fish, a high monounsaturated-to-saturated fat ratio mostly from olive oil, low-fat dairy products, and a low intake of meat and poultry [4]. This diet pattern has been shown to be protective of asthma [5–7] and allergic disease [7–9] in multiple cross-sectional observational studies. Most of these studies have been completed in Mediterranean countries where the diet is similar to the traditional pattern. In this current study, our goal was to corroborate these findings in a non-Mediterranean population of Latino children in Peru.

In Peru, asthma morbidity is a major health problem with rates above global average for reported asthma and current wheeze [10]. We compared the Peruvian diet to a Mediterranean diet pattern and investigated the relationship between the Mediterranean diet and asthma prevalence and control. We hypothesized that high adherence to a Mediterranean diet would be associated with a lower likelihood of asthma and better symptom control among children in Lima, Peru.

## Methods

### Study Setting

Participants included children enrolled in a case–control study investigating environmental and genetic associations with asthma. We recruited children 9–19 years of age who lived in the Pampas zone of San Juan de Miraflores in Lima, Peru. Asthma cases and controls were selected from a registry collected during a local census conducted by A.B. PRISMA in 2010. Eligible participants were selected at random, and the parent or legal guardian was consented and children signed an assent form. The study was approved by the Internal Review Boards at Johns Hopkins School of Medicine in Baltimore, USA and at A.B. PRISMA in Lima, Peru.

### Study Design

Participants with asthma were those who were ever diagnosed with asthma by a physician. Those with current asthma also had asthma-like symptoms (wheezing, dyspnea at rest or nocturnal dyspnea) and/or used bronchodilators, and/or inhaled corticosteroids in the past 12 months. Participants with a chronic respiratory condition other than asthma as well as those who were pregnant were excluded. Inclusion criteria for control participants included those who had never been diagnosed by a physician with asthma, had no symptoms consistent with asthma or use of asthma medications in past year, and had normal spirometry (FEV_1_ >80 % predicted and FEV_1_/FVC ratio >85 %).

### Data Collection

Questionnaires were interviewer administered to both the participant and caregiver to document asthma status and assess respiratory symptoms using standard, Spanish-translated and validated questions from the International Study of Asthma and Allergies in Childhood (ISAAC) questionnaire [11]. A health survey also collected information on 1) demographics, including age, gender, caregiver education; 2) comorbid illnesses associated with asthma (i.e., allergic rhinitis); and 3) medication use. Asthma control was assessed using the validated Spanish-translated ACT [12]. Asthma controller use was defined as having a physician prescribed inhaled corticosteroid. Current allergic rhinitis was defined as having allergic rhinitis symptoms (sneezing or a runny or blocked nose) in the last 12 months.

Height and weight were measured, and body mass index (BMI) was calculated in kg/m^2^. Our analysis used cut-off points for BMI in childhood that are based on international data and linked to the widely accepted adult cut-off points of a body mass index of 25 and 30 kg/m [13, 14]. A flow-based portable spirometer (SpiroPro, Jaeger/ERT, Hoechberg, Germany) was used to measure Forced Vital Capacity (FVC), FEV1, and FEV1/FVC. We obtained at least three acceptable and reproducible spirometric maneuvers in accordance to the American Thoracic Society (ATS) and European Respiratory Society guidelines [15]. We used height-adjusted criteria derived by Hankinson and colleagues to obtain predicted values for FEV1 and FVC [16]. We tested all patients for reversibility with 4 puffs from a salbutamol inhaler (90 mcg/puff), and reversibility was defined as increase in FEV1 of ≥12 % or an increase ≥10 % of predicted FEV1, consistent with National Asthma Education and Prevention Program (NAEPP-3) guidelines [17].

An ImmunoCAP 250 (Thermo Fisher Scientific Inc.) in vitro assay to three allergen panels (animal epidermal and proteins mix, house dust mix, and a mold and yeast mix) was used to determine atopic status. A positive result (IgE level >0.1 kUa/L) for any of the three allergen mixes was considered atopic. We had complete data to define atopic status in 76 % (*n* = 291) of our participants.

Field staff administered a FFQ, which was a Peruvian diet-specific 170-item questionnaire that assessed usual dietary intake over the previous 2 weeks, including 20 food groups and beverages. Of the 342 children with asthma, all were invited to complete the FFQ and 287 (84 %) participated. Of the 290 without asthma in the parent study, 96 (33 %) were randomly selected to complete a FFQ. The food item list was developed from 24-h recalls collected from 60 respondents who were similar in age, socioeconomic status (SES), and neighborhood to our study population. Responses include never and 1–2, 3–5, 6–9, 10–13, and 14 or more times during the last 2 weeks. A modified MDS score developed by Castro-Rodriguez et al. [18] was used, which was based on the scoring system of Psaltopoulou et al. [19]. Fruit, fish, vegetables, legumes, cereals, pasta, rice, and potatoes were considered pro-Mediterranean foods and rated according to the frequency of intake (0 points = never or occasionally; 1 point ≥1–2 times/week; 2 points ≥3 times/week) [18]. Since the study involved children, we presumed milk to be positive and not detrimental [8, 20]. Meat, sugary drinks, and fast food were considered anti-Mediterranean foods and were rated inversely (0 points ≥3 times/week; 1 point ≥1–2 times/week; 2 points = never or occasionally) [18]. Candies, industrial pastry, precooked pizzas, and fried food, along with hamburgers were classified generically as fast food [18]. Scores could range from 0 to 22 with a higher score meaning that the diet was more adherent to the Mediterranean diet.

### Statistical Analysis

Statistical analyses of differences between children with and without asthma were performed using the Chi-squared test for categorical variables and *t* test for continuous variables. Logistic regression was used to calculate odds ratios (OR) and 95 % confidence intervals. We dichotomized MDS above and below the median (15). The primary analyses investigated the relationship between MDS and asthma status (asthma vs. control) using multivariate logistic regression after adjusting for potential confounders, including age, sex, BMI category, and maternal education as an indicator of SES. Interactions between MDS and maternal education, BMI category, age and sex were tested in multivariate models. A significant interaction was found between MDS and maternal education (interaction *p* = 0.03) and multivariate models were subsequently stratified by maternal education (completed secondary education vs. did not complete secondary education).

Secondary analyses evaluated the relationship between MDS and asthma control (controlled = ACT score >19 vs. uncontrolled = ACT ≤19), atopic status, and the presence of allergic rhinitis using logistic regression and for FEV_1_ percent predicted using linear regression for the whole group and for asthmatics and controls separately. Multivariate models of asthma control did not include adjustment for asthma controller medication use as few participants utilized controller medications (2 %). There was no significant interaction between MDS and maternal education for our secondary outcomes (asthma control, atopy, allergic rhinitis, and FEV_1_) and so final models were not stratified. All analyses were performed using Stata 13.1 statistical software (Stata Corp, College Station, TX).

## Results

### Participant Characteristics

A total of 383 participants completed a FFQ, 287 asthma cases and 96 controls. Mean age was 13.5 (SD 2.6) years with no difference between those with and without asthma. Median MDS score was 15. Children with asthma were less likely to be female, more likely to have mothers who completed secondary school education, and had higher rates of atopy compared to control subjects (Table 1). Of those with asthma, 86 % had controlled asthma (ACT score >19) and only 6 (2 %) children reported having a prescribed inhaled corticosteroid.

**Table 1:** Participant characteristics

	Asthma *N* = 287	Control *N* = 96	*p* value
Female, *n* (%)	112 (39)	56 (58)	<0.01
Age, mean (SD)	13.5 (2.6)	13.4 (2.4)	0.60
BMI category, *n* (%) (*n* = 383)			0.20
Underweight	0	0	
Normal weight	176 (61)	68 (71)	
Overweight	80 (28)	22 (23)	
Obese	31 (11)	6 (6)	
Maternal education: secondary school or higher (*n* = 364)	164 (59)	33 (38)	<0.01
MDS (above median), *n* (%)	118 (41)	53 (55)	0.02
Atopic (*n* = 291)	186 (77 %)	23 (47 %)	<0.01
Current allergic rhinitis, *n* (%) (*n* = 365)	158 (57)	20 (22)	<0.01
FEV_1_ percent predicted, mean (SD)	106 (14)	110 (14)	<0.01
FEV_1_/FVC percent predicted, mean (SD)	87 (7)	91 (4)	<0.01
FEV_1_ reversibility, n (%)	45 (16)	0	<0.01
ACT score, mean (SD)	22.7 (3.2)		
% with controlled asthma (ACT >19)	86		

Comparing those who completed the FFQ with those who did not and were therefore not included in this analysis, there was a significant difference in age among controls (13.3 vs. 14.0 years, *p* = 0.04), but no significant difference in sex, BMI, or maternal education. Among those with asthma, there was no difference in asthma severity or ACT score.

### Association of Mediterranean Diet Score with Asthma Status

**Fig. 1:**
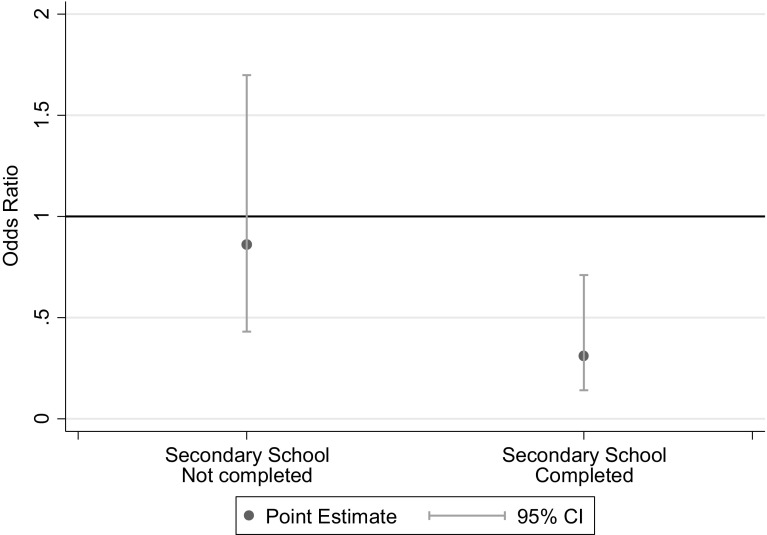
Odds of asthma if MDS is above versus below the median among children whose mother did or did not complete secondary school. Maternal secondary education completed: OR 0.31 (95 % CI 0.14, 0.71), *p* < 0.01. Maternal secondary education not completed: OR 0.86 (95 % CI 0.43, 1.7), *p* = 0.66

In bivariate analysis, being above the median MDS was associated with a decreased odds of asthma diagnosis [OR 0.56, 95 % CI (0.36, 0.90), *p* = 0.02]. In adjusted analysis, this relationship remained statistically significant, [OR 0.55, 95 % CI (0.33, 0.92), *p* = 0.02)]. A significant interaction was found between MDS and maternal education (interaction *p* = 0.03), and adjusted models were further stratified by maternal education (completed vs. did not complete secondary education). Among children whose mothers had completed secondary education, higher MDS scores were associated with a decreased odds of asthma [OR 0.31, 95 % CI (0.14, 0.71), *p* < 0.01]; however, among children whose mother had not completed secondary education, there was no statistically significant association between MDS and odds of asthma (Table 2; Fig. 1).

**Table 2:** Association between MDS and outcomes

	Crude OR (95 % CI), *p* value	Adjusted^a^ OR (95 % CI), *p* value
Asthma		
Overall group	0.56 (0.36, 0.90), *p* = 0.02	0.55 (0.33, 0.92), *p* = 0.02
High maternal education	0.33 (0.15, 0.74), *p* < 0.01	0.31 (0.14, 0.71), *p* < 0.01
Low maternal education	0.78 (0.40, 1.49), *p* = 0.45	0.86 (0.43, 1.7), *p* = 0.66
Allergic rhinitis	0.77 (0.51, 1.16), *p* = 0.21	0.75 (0.49, 1.15), *p* = 0.18
Atopy		
Overall group	0.85 (0.51, 1.42), *p* = 0.53	0.83 (0.49, 1.41), *p* = 0.49
Asthma group	0.82 (0.45, 1.49), *p* = 0.52	0.82 (0.44, 1.53), *p* = 0.53
Control group	1.3 (0.41, 3.92), *p* = 0.67	1.5 (0.45, 5.17), *p* = 0.50
Asthma control	0.78 (0.40, 1.52), *p* = 0.46	0.70 (0.35, 1.38), *p* = 0.3
FEV_1_ percent predicted		
Overall group	*β* 0.84 (−2.1, 3.8), *p* = 0.58	*β* 1.77 (−1.2, 4.7), *p* = 0.24
Asthma group	*β* 0.18 (−3.2, 3.6), *p* = 0.92	*β* 0.28 (−3.18, 3.73), *p* = 0.87

^a^Adjusted for BMI category, sex, age, maternal education; maternal education not included in models stratified by maternal education status

### Association of Mediterranean Diet Score with Asthma Control (ACT), FEV_1_, Atopy, and Allergic Rhinitis

Among those with current asthma, MDS score was not associated with asthma control status. Specifically, having an MDS above the median was not associated with greater odds of having controlled asthma (ACT >19) in bivariate [OR 0.78, 95 % CI (0.40, 1.52), *p* = 0.46] or multivariate [OR 0.70 95 % CI (0.35, 1.38), *p* = 0.3] analyses. In sensitivity analyses, after adjusting for atopic status as well as FEV_1_ percent predicted, there was still no significant effect of MDS on asthma control (data not shown). We did not find a significant relationship between MDS and FEV_1_ percent predicted, atopic status or the presence of allergic rhinitis symptoms among the group overall, or among asthma or control groups (Table 2).

## Discussion

In this study, we compared a Peruvian diet to the traditional Mediterranean diet and examined the relationship between diet pattern and asthma status. We found that among children in Peru, moderate adherence to the Mediterranean diet was associated with a lower odds of having asthma. This relationship was strongest among children whose mothers have completed secondary education. While many epidemiological studies have found a similar protective effect of a Mediterranean diet on asthma in children [5–7, 21], others have not replicated this finding [22]. Our results are consistent with the recent systematic review and meta-analysis of eight studies which showed that being in the highest tertile of MDS during childhood is a protective factor for ever being diagnosed with asthma [23].

Our Peruvian children had MDS scores that ranged from 6 to 18 (scale range 0–22) with a median of 15. These values were similar to what other studies have found using the same scale and showed that the Peruvian children studied had even slightly higher adherence to a Mediterranean diet than children studied in Spain. Garcia-Marcos used the same MDS in Spanish school children and noted a median of 13 points (range 4–20) [24]. Gonzalez Barcala also studied Spanish children and had a median of 13 (range 3–21) in the 6–7 year olds and 12 (range 4–20) in the 13–14-year-old group [22].

Adherence to a Mediterranean diet was not associated with better asthma control among those with asthma in our study. A majority of the children with asthma were controlled according to the ACT (86 %) and so the study may have been limited in detecting a difference. In Portugal, Barros et al. found that in adults, high adherence to the Mediterranean diet was associated with asthma control (defined as FEV_1_ ≥80 % of predicted, exhaled nitric oxide ≤35 ppb, and Asthma Control Questionnaire score <1) [25]. None of the available studies in children have specifically evaluated asthma control and its relationship to the Mediterranean diet pattern; however, in the systematic review of epidemiological studies assessing Mediterranean diet and prevalence of ‘current wheeze,’ there was a significant negative association with the highest compared with the lowest tertile of MDS [OR 0.85, 95 % CI (0.75, 0.98), *p* = 0.02] [23].

In our analysis, we did not find a significant relationship between MDS and atopic status or allergic rhinitis among the asthma or control groups. Similar to our results, the ISAAC (*n* = 50,004) did not find an association between MDS and allergic sensitization [6]; however, other results suggest that MDS adherence may be related to decreased prevalence of atopy or allergic rhinitis. In Crete, Chatzi et al. noted that adherence to the Mediterranean diet was associated with a decreased odds of allergic rhinitis [8]. Similarly, De Batlle et al. found a negative relationship between being in the two higher tertiles of MDS and allergic rhinitis ever, current sneezing, and itchy-watery eyes [7].

Maternal education was used as a marker of SES, and though it is not clear why adherence to a Mediterranean diet is only protective of asthma in children with more educated mothers, there are several plausible explanations. Perhaps these parents made overall healthier diet and lifestyle choices for their families, or children of mothers with less education may have other factors common in environments of lower SES that outweigh the relevance of diet in contributing to asthma development, such as possible increased exposure to pollution and increased stress, which are recognized contributors to asthma morbidity [26–29].

There are no Mediterranean diet intervention trials in children, and studies supplementing the diet with antioxidants have had mixed results [30, 31]. In a 12-week open-label trial by Sexton et al., 38 adults with current asthma were randomized to a high intervention group (41 h of dietician services), low intervention group (2 h of services), or control (one dietician session and recipes) [32]. The study achieved its primary outcome of altering the eating habits of participants in the high intensity intervention toward a Mediterranean diet pattern [32]. The study was not powered to detect clinical endpoints but did note statistically non-significant improvements in asthma-related quality of life and spirometry in the intervention groups [32]. There was no effect on asthma control [32].

Our study had several limitations. The FFQ can be associated with recall bias and as with all cross-sectional studies; we are unable to show the time relationships of the associations found, so it is not possible to reach causal conclusions. While there is no validated FFQ in our population, the current FFQ was developed from 24 h recalls collected in our Peruvian community. This FFQ is effective at ranking participants relative to each other in terms of the intake frequencies that were used to calculate MDS. The FFQ does not allow correction for energy intake, but there was no difference in BMI categories between those with and without asthma. Furthermore, dietary scores estimating adherence to a Mediterranean diet have been developed, but there is no validated MDS available for use in children. Our scoring system and analysis of the MDS was a modified one used in pediatric populations in other studies [6, 18, 22, 24].

## Conclusions

Our results show an inverse association between adherence to a Mediterranean diet and odds of asthma among children in Lima, Peru suggesting a protective role of Mediterranean diet on the development of asthma. We did not find an association between high adherence to the Mediterranean diet and level of disease control, FEV_1_, allergic rhinitis, or atopic status. In conclusion, diet may be an important, modifiable risk factor for the development of asthma in children, and the present findings need to be confirmed by interventional or prospective studies prior to recommending intake of the Mediterranean diet for asthma prevention or treatment.

## Abbreviations

MDS: Mediterranean diet score
FFQ: Food frequency questionnaire
BMI: Body mass index
ACT: Asthma control test
FEV_1_: Forced expiratory volume in one second
FVC: Forced vital capacity
ISAAC: International Study of Asthma and Allergies in Childhood
ATS: American Thoracic Society
OR: Odds ratio
SES: Socioeconomic status
NAEPP: National Asthma Education and Prevention Program
